# Predictive factors for histological response to neoadjuvant therapy in gastric adenocarcinomas

**DOI:** 10.1590/0102-67202025000043e1912

**Published:** 2025-12-19

**Authors:** Dhouha BACHA, Nour BOUDRIGUA, Ines MALLEK, Safé CHAMMEM, Monia ATTIA, Lassaad GHARBI, Ahlem LAHMAR, Sana BEN-SLAMA

**Affiliations:** 1 Mongi Slim University Hospital, Pathology Department – La Marsa, Tunis, Tunisia.; 2 University Tunis El Manar, Faculty of Sciences of Tunis, Laboratory of Genetics, Immunology, and Human Pathologies – Tunis, Tunisia.; 3 Mongi Slim University Hospital, Radiology Department – La Marsa, Tunis, Tunisia.; 4 Mongi Slim University Hospital, Visceral Surgery Department – Tunis, Tunisia.

**Keywords:** Stomach, Carcinoma, Neoplasm Regression, Spontaneous, Histology, Prognosis, Estômago, Carcinoma, Regressão Neoplásica Espontânea, Histologia, Prognóstico

## Abstract

**Background:**

Perioperative chemotherapy is the standard curative treatment for resectable gastric adenocarcinoma, significantly improving both overall and recurrence-free survival. The histological response to neoadjuvant therapy is a critical prognostic factor, commonly assessed through grading systems such as Mandard’s tumor regression grade (TRG).

**Aims:**

The aim of the study was to identify predictive factors for histological response to neoadjuvant therapy in gastric adenocarcinoma.

**Methods:**

A retrospective study was performed on patients with gastric adenocarcinoma who underwent surgery following neoadjuvant chemotherapy, from 2015 to 2020. The histological response was evaluated using Mandard TRG, which includes five grades (1–5), based on the proportion of residual viable tumor cells and fibrosis. Grades 1–3 were considered a response, and Grades 4 and 5 were considered no response. Students’ t-test, chi-squared test, and multivariate logistic regression were used, with significance set at p<0.05.

**Results:**

Forty patients were included (male-to-female ratio 2.64, mean age 63 years). Histological response (TRG 1–3) was observed in 48%, while 52% showed no response (TRG 4–5). Univariate analysis showed significant correlations between histological response and tumor size >38 mm (p=0.03), differentiation (p=0.02), parietal wall invasion, absence of nodal involvement (both p<0.001), pathological tumor, node, and metastasis stage (p<0.001), and absence of vascular and perineural invasion (both p=0.001). Multivariate analysis identified parietal wall invasion (odds ratio=2.351, p=0.022) and absence of lymph node metastases (odds ratio=1.491, p=0.01) as independent predictive factors.

**Conclusions:**

Parietal wall invasion and absence of nodal metastases are predictive of histological response to neoadjuvant therapy in gastric adenocarcinoma.

## ARTICLE HIGHLIGHTS

Perioperative chemotherapy is the standard curative treatment for resectable gastric adenocarcinoma, and histological response is a key prognostic factor.

In this retrospective Tunisian cohort of 40 patients, histological response [Mandard tumor regression grade (TRG) 1–3] was achieved in 48%, while 52% showed no response (TRG 4–5).

Multivariate analysis identified parietal wall invasion and lymph node metastases as independent predictors of poor histological response.

Patients with histological response had significantly better overall survival, confirming the prognostic value of TRG in gastric cancer management.

## Summary

**CENTRAL MESSAGE:**

Currently, perioperative chemotherapy (CT) is the standard curative treatment for resectable forms of gastric carcinoma. This approach significantly improves both overall survival and recurrence-free survival. A key prognostic factor in evaluating therapy effectiveness is the histological response to neoadjuvant CT, which pathologists assess in gastric resection specimens using various grading systems. Notable examples include tumor regression grading by Mandard and the Becker grading system.

**PERSPECTIVES:**

Identifying predictive factors before surgery is crucial for guiding clinical decision-making and avoiding ineffective neoadjuvant treatment with its associated toxicity. Personalized treatment planning, informed by individual tumor biology and anticipated response, represents a major step toward optimizing outcomes in patients with locally advanced gastric adenocarcinoma.

## VISUAL ABSTRACT

**Figure:**
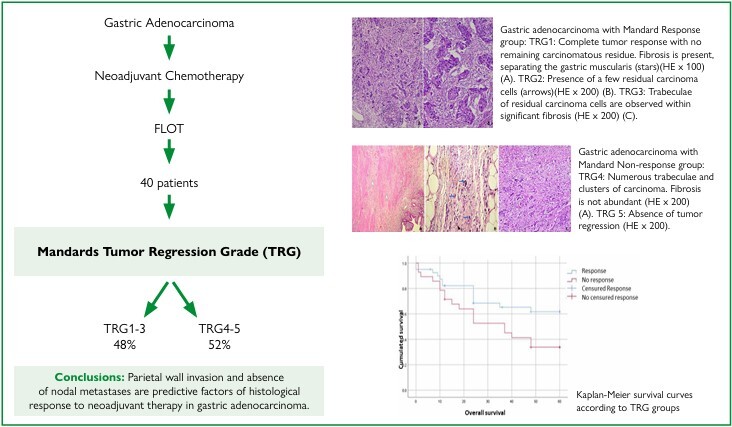

## RESUMO

**Racional:**

A quimioterapia perioperatória é o tratamento padrão para o adenocarcinoma gástrico ressecável, melhorando a sobrevida global e livre de recidiva. A resposta histológica à terapia neoadjuvante, avaliada pelo grau regressão tumoral (GRT) de Mandard, é um importante fator prognóstico.

**Objetivos:**

Identificar fatores preditivos para resposta histológica à terapia neoadjuvante no carcinoma gástrico.

**Métodos:**

Estudo retrospectivo com pacientes submetidos à cirurgia após quimioterapia neoadjuvante (2015–2020). A resposta foi classificada em GRT 1–3 (com resposta) e GRT 4–5 (sem resposta). Foram aplicados testes estatísticos, com p<0,05 como nível de significância.

**Resultados:**

Foram analisados 40 pacientes (razão H/M 2,64; média de idade 63 anos). Resposta histológica completa (GRT 1–3) foi observada em 48%, enquanto 52% não apresentaram resposta (GRT 4–5). A análise univariada mostrou associação significativa com: tumor >38 mm (p=0,03), diferenciação (p=0,02), invasão parietal, ausência de linfonodos acometidos (ambos p<0,001), estadiamento pTNM (p<0,001), e ausência de invasão vascular/perineural (p=0,001). Na análise multivariada, invasão parietal (*odds ratio*=2,351; p=0,022, p<0.05) e ausência de metástases linfonodais (*odds ratio*=1,491; p=0,01, p<0.05) foram preditores independentes.

**Conclusões:**

A invasão parietal e a ausência de metástases linfonodais são fatores preditivos de resposta histológica à quimioterapia neoadjuvante no adenocarcinoma gástrico.

## INTRODUCTION

 Gastric carcinoma (GC) remains a major public health concern, ranking as the fifth most common cancer globally and the fourth leading cause of cancer-related mortality^12^. In Tunisia, GC ranked eighth among diagnosed cancers and was the fourth leading cause of cancer deaths, accounting for 7.7% of mortality-related cancer^9^. Currently, perioperative chemotherapy (CT) is the standard curative treatment for resectable forms of GC. This approach significantly improves both overall survival (OS) and recurrence-free survival (RFS)^13^. A key prognostic factor in evaluating therapy effectiveness is the histological response to neoadjuvant CT, which pathologists assess in gastric resection specimens using various grading systems^2^. Notable examples include tumor regression grade (TRG) by Mandard^15^ and the Becker grading system^3^. These grading systems highlight the importance of identifying factors that influence the CT response. Recognizing these factors can help stratify patients based on their responses, allowing for early adjustments in therapeutic strategies. This study aimed to identify predictive factors for the histological response to neoadjuvant therapy in GC, specifically using the Mandard classification. 

## METHODS

 This was a retrospective, descriptive, single-center study conducted over a 5-year period, from January 2015 to January 2020, at our institution. Eligible patients were those with histologically confirmed gastric adenocarcinoma (ADC), classified according to the 2019 World Health Organization (WHO) criteria, and who underwent surgical resection following neoadjuvant CT^16^. Exclusion criteria included primary tumors originating from adjacent organs with secondary invasion of the stomach, patients whose diagnosis was based solely on endoscopic biopsy without subsequent surgical intervention, and those with incomplete or unusable medical records. 

 Clinical, endoscopic, therapeutic, pathological, and follow-up data were extracted from electronic medical records and pathology reports. Macroscopic tumor characteristics were classified according to Bormann’s classification^4^. Clinical staging of tumor, node, and metastasis (cTNM) was performed based on physical examination, imaging studies, and biopsy results prior to any treatment, using the eighth edition of the tumor, node, and metastasis (TNM) classification published by the Union for International Cancer Control in 2017^5^. 

 The histological tumor response to neoadjuvant CT was assessed using the Mandard TRG system, which ranges from grade 1 (complete regression) to grade 5 (no regression), based on the extent of fibrosis relative to the proportion of viable tumor cells^15^. 

 For statistical analysis, TRG scores were grouped into two categories: Response (TRG 1–3) and no response (TRG 4–5). All data were entered and analyzed using the Statistical Package for the Social Sciences, Windows version. Associations between clinical, endoscopic, and pathological variables and histological response were analyzed using Pearson’s χ^2^ test. Fisher’s exact test was applied when expected cell counts were below five. 

 The study also analyzed prognostic factors, including age, sex, clinical stage (cTNM), tumor size, histological grade, presence of vascular emboli (VE), and perineural invasion (PNI). Overall survival (OS) was assessed using Kaplan-Meier survival curves, with the date of surgery defined as the starting point. Univariate analysis was used to identify survival predictors, and survival distributions were compared using the log-rank test. The same variables used in the correlation analyses were included. As this was a retrospective study using previously collected data, informed consent was not required. Patient anonymity was maintained throughout the study. No conflicts of interest were declared. 

## RESULTS

 A total of 40 patients were included in the study. The mean age of the patients was 61.9 years ±9.8. The median age was 63 years, with ages ranging from 40 to 77 years. Among the patients, 29 were male and 11 were female, resulting in a male-to-female ratio of 2.64:1. All cases in our study were diagnosed as gastric ADC. 

**Figure 1:**
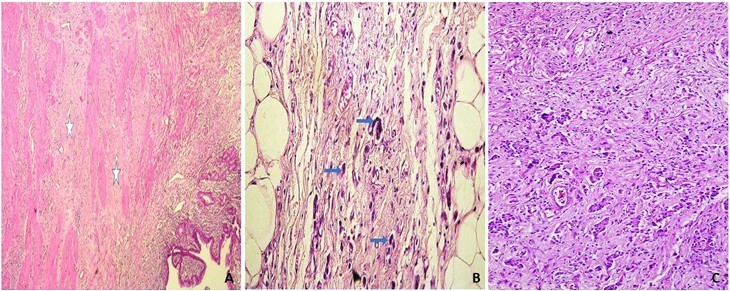
Gastric adenocarcinoma with Mandard analysis: (A) TRG1: Complete tumor response with no remaining carcinomatous residue. Fibrosis present, separating the gastric muscularis (stars) (HE ×100). (B) TRG2: Presence of some residual carcinoma cells (arrows) (HE ×200). (C) TRG3: Trabeculae of residual carcinoma cells are seen amidst significant fibrosis (HE ×200).

**Figure 2:**
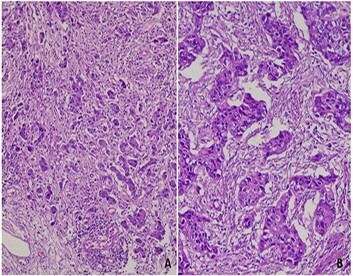
Gastric adenocarcinoma with Mandard analysis. Non-response group: (A) TRG4: Numerous trabeculae and carcinoma clusters. Fibrosis is not abundant (HE ×200). (B) TRG5: Absence of tumor regression (HE ×200).

 Neoadjuvant therapy consisted of four cycles of FLOT (5-fluorouracil, leucovorin, oxaliplatin, and docetaxel) CT administered to all patients. Additionally, six patients received neoadjuvant radiotherapy (RT) at a dose of 45 Gy delivered in 25 fractions over 5 weeks. RT was combined with a radiosensitizing CT regimen, typically continuous infusion of 5-FU (5-fluorouracil). Postoperatively, all patients underwent four additional cycles of FLOT CT. Adjuvant RT was administered to 10 patients, with a total dose ranging from 45 to 50.4 Gy in 25–28 fractions, combined with intravenous 5-FU. In our series, 48% of patients were classified in the response group (Figure 1), while 52% were in the non-response group (Figure 2). 

TRG: tumor regression grade.

TRG: tumor regression grade.

 The clinicopathological and therapeutic characteristics of the patients in our study are illustrated in Table 1. 

**Table 1:** Clinicopathological and therapeutic characteristics of the patients in our study.

Parameters		n (%)
**Symptoms**		
	Epigastric pain	34 (85)
	General health deterioration	25 (63)
	Nausea/vomiting	11 (28)
	Gastrointestinal bleeding	7 (18)
	Dysphagia	5 (13)
	Chronic anemia	8 (20)
**Bormann endoscopic appearance**		
	Ulcerative	20 (50)
	Mass	14 (35)
	Infiltrative ulcerative	6 (15)
**Tumor location**		
	Proximal Stomach	18 (45)
	Distal Stomach	22 (55)
**Type of gastrectomy**		
	Total gastrectomy	32 (80)
	Subtotal (4/5) gastrectomy	8 (20)
**Surgical resection**		
	R0	36 (90)
	R1	4 (10)
**Tumor grade**		
	Low grade	24 (60)
	High grade	16 (40)
**Vascular emboli**		
	Yes	17 (42.5)
	No	23 (57.5)
**Perineural invasion**		
	Yes	13 (33)
	No	27 (67.5)
**Neoadjuvant therapy**		
	Chemotherapy	34 (85)
	Radiochemotherapy	6 (15)
**Adjuvant therapy**		
	Chemotherapy	31 (77.5)
	Radiochemotherapy	9 (22.5)
**Stage cTNM**		
	IIA	14 (35)
	IIB	9 (22.5)
	IIIA	8 (20)
	IIIB	1 (3)
	IIIC	5 (12.5)
	IV	3 (1)
**Mandard tumor regression grade**		
	TRG 1	4 (10)
	TRG 2	6 (15)
	TRG 3	9 (23)
	TRG 4	18 (45)
	TRG 5	3 (7)
**Histological response category**		
	Reponse group	19 (48)
	No reponse group	21 (52)

TNM: tumor, lymph nodes, and metastasis; R0: complete cancer removal; R1: microscopically incomplete tumor removal; TRG: tumor regression grade.

### Association between histological response categories and pre-therapeutic clinico-pathological features

 This association, in univariate analysis, was illustrated in Table 2. When comparing the tumor regression response groups (TRG 1–3 vs. TRG 4–5), no significant association was found with age (p=0.785, p>0.05). However, a tumor size of 38 mm was significantly associated with a better response (TRG 1–3) compared to tumors >38 mm (p=0.003, p<0.05). Histological differentiation also showed a significant correlation, with well and moderately differentiated tumors responding better than poorly differentiated ones (p=0.002, p<0.05). The absence of VE and PNI was significantly associated with tumor regression (p=0.001, p<0.05, for both). 

**Table 2:** Univariate analysis to identify the association of histological response categories and clinico-pathological parameters in patients of our study.

Parameters		Response group (TRG 1–3)	No response group (TRG 4–5)	p-value
**Age (Years)**				0.785
	=60	6	9	
	>60	13	12	
**Tumor size (mm)**				0.003
	=38	13	8	
	>38	6	13	
**Tumor differentiation**				0.002
	Well differentiated	7	6	
	Moderately differentiated	6	5	
	Poorly differentiated	6	10	
**Vascular emboli**				0.001
	Yes	4	13	
	No	15	8	
**Perineural invasion**				0.001
	Yes	3	10	
	No	16	11	
**Parietal invasion**				<0.001
	cT3–cT4	9	17	
	cT1–cT2	10	4	
**Lymph node metastasis**				<0.001
	cN0–cN2	18	17	
	cN3	1	4	
**Stages**				<0.001
	Early stage (II)	14	9	
	Advanced stages (III+IV)	5	12	

N: lymph nodes; T: tumor stage; TRG: tumor regression grade.

 Additionally, patients with superficial parietal invasion (cT1–cT2) showed a significantly better response than those with deep invasion (cT3–cT4) (p<0.001). The presence of lymph node metastasis at stage cN3 was significantly associated with poor response (p<0.001). Finally, patients at stage II had significantly better regression compared to those with advanced stages (III and IV) (p<0.001). 

 Multivariate logistic regression analysis identified two independent predictors of poor tumor regression. Parietal wall invasion was significantly associated with lower response to treatment, with an odds ratio (OR) of 1.351 (95% confidence interval (CI) 1.033–1.776; p=0.022, p<0.05). Similarly, the presence of lymph node metastasis was strongly correlated with poor response, showing an OR of 1.491 (95%CI 1.1711.888; p=0.001, p<0.05). 

### Survival outcomes

**Figure 3:**
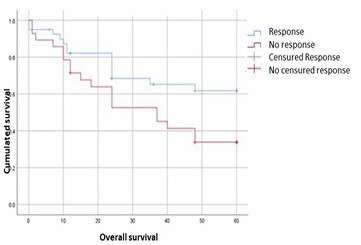
Kaplan-Meier survival curves according to the tumor regression grade groups.

 The mean OS in our cohort was 39.91±20.58 months. The 1, 3, and 5-year survival rates were 78, 57, and 49%, respectively. Thirteen patients (33%) died during the follow-up period, with a mean time to death of 21.07±13.67 months. When stratifying patients by histological response, OS was significantly higher in the response group, with a mean OS of 44.37 months, compared to 34.10 months in the non-response group (p=0.002, p<0.05, log-rank test). This result highlights the prognostic value of TRG following neoadjuvant therapy. Kaplan-Meier survival curves according to TRG groupings are shown in Figure 3. 

## DISCUSSION

 This study aimed to identify predictive factors of histological response to neoadjuvant therapy in gastric ADC using the Mandard TRG and to evaluate the prognostic value of this response on OS. Among the 40 patients included, 48% were in the response group (TRG 1–3). Univariate analysis revealed significant associations between tumor regression and several pre-therapeutic variables, including tumor size >38 mm, poor degree of differentiation, deeper parietal invasion (cT3–cT4), cN3 lymph node metastases, vascular and PNI, and advanced clinical stages (III and IV). 

 Multivariate logistic regression analysis demonstrated that both deeper parietal invasion and nodal involvement are significant risk factors for reduced histological tumor regression following neoadjuvant therapy. Furthermore, the analysis of survival outcomes demonstrated that histological response was significantly associated with OS (p=0.002, p<0.05), with better survival rates observed in patients with the response group. These findings support the role of histological tumor regression as both a predictive and prognostic marker in the neoadjuvant setting. 

### TRG systems

 In gastric cancer, as in other types of cancer, several classification systems have been proposed. Among them are the Japanese Gastric Cancer Association classification^7,8^, the College of American Pathologists system^6^, the China-TRG system^24^, the Becker system^3^, and the Mandard TRG^15^. However, none of these classifications has been consensually validated for use in gastric cancer. 

 The Mandard TRG was published in 1994 and was initially applied to evaluate tumor regression in esophageal squamous cell carcinomas following neoadjuvant treatment with cisplatin and RT^15^. According to the study by Xu et al.^22^ involving 264 patients with advanced gastric ADC, tumor response results following neoadjuvant CT showed a predominance of TRG 3 (38.3%) compared to the other grades. TRG 1 was the least frequent (7.2%, n=19). The distribution of the remaining grades was as follows: TRG 2 in 60 patients (22.7%), TRG 4 in 80 patients (30.3%), and TRG 5 in 4 patients (1.5%)^14^. The study by Wang et al.^21^ also showed a predominance of TRG grade 3 (37%, n=40), while grade 5 was the least represented (5.6%, n=6). In our series, TRG grade 4 was the most frequent (45%), while grade 5 was the least represented (7%). 

 These results partially contrast with studies such as Wang et al.^21^ and Xu et al.^22^, which reported TRG 3 as the most common response grade. This difference may reflect variability in patient populations, treatment protocols, or interpretation of histological regression^18,19^. 

 These inter-study differences in TRG distribution highlight the variability in histological response assessment across different clinical settings. Such discrepancies may stem from differences in patient selection, tumor biology, neoadjuvant regimens, or even interobserver variability in pathological interpretation^2^. This underscores the need for standardization in TRG assessment and suggests that future studies should aim to correlate regression patterns with specific clinical or molecular profiles to enhance the reproducibility and prognostic value of histological response grading. Tumor differentiation and size were significantly associated with response, consistent with findings by Wang et al.^21^, who reported that well-differentiated and smaller tumors respond more favorably to treatment. Furthermore, the presence of vascular and PNI was associated with poorer outcomes, supporting their role as negative predictive markers. 

### Tumor regression and survival

 The prognostic value of TRG has been confirmed in several studies. In the study by Xu et al.^22^, a significant association was observed between TRG and OS (p<0.001), which is consistent with our findings (p=0.002, p<0.05). Although this difference did not reach statistical significance (p=0.535, p>0.05)^5^, the trend further supports the prognostic relevance of histological response. These observations reinforce the notion that tumor regression after neoadjuvant therapy can serve as a surrogate marker for survival. Moreover, the absence of response not only portends a poorer prognosis but may also expose patients to the unnecessary toxicity of CT without therapeutic benefit^6^. This highlights the need for predictive markers that can better identify likely responders before initiating neoadjuvant treatment. 

### Predictive factors of response

 About the correlation between tumor regression and several clinico-pathological factors, the literature series is partially consistent with our results. Regarding histological differentiation, Wang et al.^21^ identified it as a key predictive factor, showing that well-differentiated tumors were more likely to respond to neoadjuvant CT. Interestingly, Liang et al.^11^, in a large cohort of 867 patients, observed better responses in poorly differentiated tumors and early-stage disease (IB–IIA), highlighting potential biological variability in response patterns. 

 Tumor size also emerged as a significant factor in several studies. Wang et al.^21^ reported that smaller tumors were more responsive to neoadjuvant therapy. Tumor size emerged as an independent predictor of response, with Japanese studies highlighting this association particularly in Bormann type IV tumors exceeding 7 cm^17,20^. 

 Lymph node involvement, a major predictor in our series, was also emphasized by Xu et al.^22^ and Yonemori et al.^23^. The latter additionally reported a correlation between baseline hemoglobin level and treatment response. 

 Tumor location has been investigated with mixed findings. Liang et al.^11^ observed better response rates in proximal tumors compared to distal ones (71.08 vs. 42.18%), though the difference was not statistically significant. Similarly, Ajani et al. found no association between tumor location and response to paclitaxel-based CT^1^. However, in the nomogram proposed by Liang et al.^11^, tumor location was included alongside other predictive variables such as smoking status, depth of invasion, nodal status, and differentiation. 

 Lorenzen et al.^14^, in a retrospective analysis, found that tumors located in the upper two-thirds of the stomach were associated with improved response rates, a finding further supported by Li et al.^10^. Collectively, these results indicate that tumor regression following neoadjuvant therapy is influenced by multiple factors, including tumor biology, anatomical location, and disease extent. The development of standardized predictive models that incorporate these variables may contribute to more personalized and effective therapeutic strategies. 

 Treatment adaptation in the presence of factors associated with poor response to CT may involve intensifying this treatment. Conversely, certain CT side effects can be avoided in "good" responders with a personalized, relatively less aggressive treatment approach. A summary of key studies evaluating predictive factors of response to neoadjuvant therapy in gastric cancer is illustrated in Table 3. 

**Table 3:** Summary of key studies evaluating predictive factors of response to neoadjuvant therapy in gastric cancer.

Authors (Year)	Country	Sample size	Main predictive findings
Ajani et al. (2005)^1^	USA	41	No association between response and age, sex, tumor location, TNM stage, or histological type.
Li et al. (2012)^10^	China	73	Proximal tumors responded better; perioperative FOLFOX was associated with better survival and tolerance.
Liang et al. (2023)^11^	China	867	Poorly differentiated, proximal tumors and early-stage disease showed better response; a predictive nomogram was developed. MSI status is not predictive.
Lorenzen et al. (2012)^14^	Germany	410	Tumor location in the upper two-thirds of the stomach was associated with better response.
Tsuburaya et al. (2014)^20^	Japan	51	Bormann type IV tumors >7 cm responded more favorably to neoadjuvant therapy.
Wang et al. (2012)^21^	South Korea	108	Tumor differentiation and size were independent predictors of tumor regression; better OS was observed in responders.
Yonemori et al. (2004)^23^	Japan	119	Hemoglobin level and lymph node metastases were associated with treatment response.

TNM: tumor, lymph nodes, and metastasis; OS: overall survival; FOLFOX: folinic acid (leucovorin), fluorouracil (5FU), and oxaliplatin; MSI: microsatellite instability.

### Strengths and limitations

 The involvement of the same pathologist ensured consistency in histological review. Furthermore, the study specifically targets a Tunisian population, which is often underrepresented in the literature. Several limitations of our study include the small sample size (40 patients), its retrospective design, and the lack of strict standardization in the therapy protocol. 

 Furthermore, although cTNM provides valuable preoperative information, its accuracy, particularly in assessing lymph node involvement and local tumor extension, is limited when compared to the pathological tumor, node, and metastasis (pTNM) classification. The latter, based on histological examination of the resected gastric specimen, offers a more precise staging. However, in our context, the pTNM classification could not be used to identify predictive factors of treatment response, as the surgical specimen had already been exposed to neoadjuvant therapy, which alters the native histological characteristics. To address this limitation, future studies should integrate both pre- and post-treatment staging data and, when possible, include radiological-pathological correlation or biomarker-based predictive models to enhance the accuracy of treatment response assessment. 

### Clinical implications

 Based on our findings, several practical recommendations can be proposed to improve pre-therapeutic evaluation in patients with gastric ADC. Accurate assessment of parietal wall invasion, lymph node involvement, and tumor size is essential, as these factors significantly influence the response to neoadjuvant therapy. This assessment should rely on high-resolution imaging modalities such as abdominal CT and, preferably, endoscopic ultrasound, which offers superior accuracy for local staging. Moreover, gastric biopsies should be multiple and of adequate depth and volume to reliably evaluate tumor differentiation and detect VE, both of which were shown to be associated with histological response. Implementing these measures could help identify patients most likely to benefit from neoadjuvant therapy and optimize individualized treatment planning. 

 In our study, histological response to neoadjuvant therapy, evaluated using the Mandard TRG, was significantly associated with pre-therapeutic gastric wall invasion and regional lymph node metastases. These findings are partially consistent with previous studies, which have also highlighted the potential influence of factors such as smoking status, hemoglobin levels, and tumor location. 

 From a clinical perspective, these insights support the development of more adaptive therapeutic strategies, allowing for early identification of poor responders who may benefit from prompt surgical intervention. This approach not only minimizes unnecessary CT toxicity but also improves individualized care. 

## CONCLUSIONS

 Identifying predictive factors before surgery is crucial for guiding clinical decision-making and avoiding ineffective neoadjuvant treatment with its associated toxicity. Personalized treatment planning, informed by individual tumor biology and anticipated response, represents a major step toward optimizing outcomes in patients with locally advanced gastric ADC. 

 In the future, the integration of predictive scoring systems, based on both clinical and biological variables, may enhance our ability to tailor treatment. Multicenter studies with larger cohorts will be essential to validate these predictors and develop robust, reliable tools to guide treatment decisions. 

## Data Availability

The Informations regarding the investigation, methodology and data analysis of the article are archived under the responsibility of the authors.
